# Cultural and educational environment in the development of younger schoolchildren’s creative potential

**DOI:** 10.3389/fpsyg.2023.1178535

**Published:** 2023-09-15

**Authors:** Vera Yu. Khotinets, Evgeniya O. Shishova

**Affiliations:** ^1^Department of General Psychology, Institute of Pedagogy, Psychology and Social Technologies, Udmurt State University, Izhevsk, Russia; ^2^Department of Educational Psychology, Institute of Psychology and Education, Kazan (Volga Region) Federal University, Kazan, Russia

**Keywords:** cultural-historical psychology, cultural-educational environment, educational environment design, creative potential, communicative creativity, primary school ages

## Abstract

The purpose of our research is to study the creative potential as psychological capacities for younger schoolchildren’s creative self-realization and self-development in various conditions of the educational environment. The methodological basis of this work is Vygotsky’s conceptual provisions according to which the human psyche is culturally determined, and a sociocultural environment is considered to be the main source and condition for the child’s mental development. The study involved younger schoolchildren (a total of 160 children from the 4th grade aged 9–10 years, *n* = 160, *M* = 9.5 years, SD = 2.6; 49% boys) from schools in Kazan (Russian Federation). We used a test of verbal creativity when studying the creative potential of younger schoolchildren, the proposed method is a Russian-language adapted version of the RAT test (remote association test) by Mednik. The Johnson Creativity Inventory was used as adapted by Tunick. To study the level of communicative control, the test “Diagnostics of communicative control” by Schneider was used. To assess the personal qualities of younger students, we used a modified version of the children’s personality questionnaire intended for 8–12 year-old children and developed by Cattell and Koan. As a result of a comprehensive expert assessment, we identified four types of schools with different severity degrees of essential characteristics of educational environments: serene, dogmatic, career and creative. According to the analysis of variance (one-dimensional one-factor ANOVA), the younger schoolchildren’s creative potential was revealed in the context of the educational environment variability and the contingency of the educational environment parameters with the personal characteristics of the children. We have empirically confirmed that in a *creative* educational environment with cultural content based on ethno-cultural values, patterns and norms, the development of the child is actively supported largely, with the disclosure of his creative potential. Younger schoolchildren are characterized by greater subjective agency and the capability to gain unique achievements in educational and cognitive activity.

## 1. Introduction

**Research problem.** One of the focuses in current research is the problem of creating a system of conditions for the personality formation. This system provides positive opportunities and various options for choosing the optimal trajectory of the personality development, which places the concept of “cultural and educational environment” among the basic ones in a modern developmental education. **An educational environment** is studied as a component of the social situation of the child mental development and as a condition for its personal development (Leontyev, 1975; Vygotsky, 1999, 2005; Yasvin, 2010; Veraksa, 2018; Veraksa et al., 2019; Rubtsov and Ulanovskaya, 2022 and others). However, the research into the influence that educational systems exert on the child’s intellectual, emotional and personal development primarily focuses on the consideration of theoretical aspects, there are very few empirical studies of the educational environment developing potential, which is specific to each educational institution. Existing studies, devoted to this problem, are very contradictory, they do not take into account the current reforms in the field of education (Rubtsov and Ulanovskaya, 2022). In this regard, of primary importance for educational psychology is the problem of assessing the quality of education in educational institutions that provide specific conditions and development opportunities for the subjects of education. This accounts for **the significance** of the research into the creative potential of a growing person, the need to further explore the social situation of development and the conditions for the ontogenesis of creativity, potentially contributing to its formation. By **a cultural and educational environment** we mean a system of conditions and opportunities for the development of subjects of education with cultural content (Khotinets and Medvedeva, 2021). **Creative potential at primary school age** is understood as an integrative quality, reflecting the measure of the younger schoolchild’s creative self-realization and self-development ability (Veraksa, 1990). Primary school age is the period most open to various changes. A change in the leading activity promotes “the erasure” of past experiences, laying a new foundation for the child’s personality. During this period, the younger schoolchild is most sensitive to the formation of a cognitive attitude to the world, the manifestation of free personal expression, the development of creative abilities, and communicative creativity, which ensures the creative nature of communication and communicative activity of the child (Runco and Acar, 2012; Runco et al., 2020; Khotinets et al., 2022; Shishova and Akhatova, 2022 etc.). According to Vygotsky’s theoretical provisions concerning the systemic nature of the higher mental functions’ development, at the early school age, thinking becomes a “system-forming” function moving from the visual-figurative to its verbal-logical type, which undoubtedly affects other mental functions seeking to occupy the center of consciousness. The change in the system of internal relationships allows the central function to become more differentiated and developed. At this time, other mental processes function as processes serving the formation of the central function. Thus, the complexity of interfunctional relationships and the differentiation of mental functions gradually increase. To acquire higher mental functions, it is necessary to transfer and assimilate knowledge about their structures in an organized educational environment through specially organized training (Vygotsky, 1999).

In the context of studying a growing person’s creative potential, the problems identified by Vygotsky remain relevant today: “the relationship between learning and development at school age,” “the social situation of development,” “mechanisms for the practical mastery of reality.” According to Vygotsky, it is “learning that creates the zone of proximal development, that is, it brings up the child’s interest in life, awakens and sets in motion a whole series of internal development processes that are so far possible for the child only in the sphere of its relationships with others and through its cooperation with peers, but which, performing the internal course of development, later become the child’s own internal property” (Vygotsky, 1935, p. 16). The “social situation of development” is understood as “a completely peculiar, specific for a given age, exclusive, unique and inimitable relationship between the child and the reality surrounding it, primarily the social one” (Vygotsky, 1984, p. 258). This social reality is “the main source of development” when the social becomes the individual.

### 1.1. Literature review

According to Vygotsky, “in the child’s development, the outcomes that we are to achieve at the end of the development, as a result of this development, are already given in the environment from the very beginning” (Vygotsky, 2001, p. 83). “The greatest feature of the child’s development is that this development takes place in such conditions of interaction with the environment, when the ideal form, the final (cultural) form that should appear at the end of development, not only exists in the environment and comes into contact with the child from the very beginning, but also actually interacts with it, influencing the primary (natural) form, the first steps of the child’s development, i.e., something that should take shape at the very end of the development somehow influences the very first stages of this development” (Vygotsky, 2001, pp. 83–84).

Answering to the question about the role of the educational environment in the mental and personal development of the child, Vygotsky said that “in relation to the development of higher human-specific properties and forms of activity, the environment acts as a source of this development, i.e., it is the interaction with the environment that is the source generating these properties in the child” (Vygotsky, 2001, p. 88).

According to Vygotsky, “the best stimulus for children’s creativity is the organization of their life and environment in such a way that it creates the needs and opportunities for children’s creativity” (Vygotsky, 2004, pp. 57–58). The meaning and significance of children’s creativity lies in the fact that it allows the child to overcome that tough challenge in the development of creative imagination, which gives a new and lifelong direction to his fantasy. The meaning of children’s creativity is its effect of deepening, expanding and cleansing of the child’s emotional life. The significance of children’s creativity is its ability to allow the child, by exercising its creative aspirations and skills, to master human speech - the most subtle and complex instrument for the formation and transmission of human thoughts, human feelings, human inner world (Vygotsky, 2004, pp. 60–61).

Vygotsky highlights the importance of cultivating creativity at school age. A person can comprehend his whole future with the help of creative imagination. His orientation in the future, his behavior, based on the future and proceeding from this future, is the main function of the imagination. And since pedagogical work is mainly oriented toward preparing the students’ behavior for the future, the development and exercise of their imagination are the main driving forces in realizing this goal. The shaping of a creative personality, aspiring to the future, is prepared by creative imagination embodied in the present (Vygotsky, 2004, p. 78).

This report argues that a national strategy for creative and cultural education is essential to that process. We put the case for developing creative and cultural education; we consider what is involved; we look at current provision and assess the opportunities and obstacles; and we set out a national strategy. By creative education we mean forms of education that develop young peopleʻs capacities for original ideas and action: by cultural education we mean forms of education that enable them to engage positively with the growing complexity and diversity of social values and ways of life. We argue that there are important relationships between creative and cultural education, and significant implications for methods of teaching and assessment, the balance of the school curriculum and for partnerships between schools and the wider world (The National Advisory Committee on Creative and Cultural Education (NACCCE), 1999).

This report argues that a national strategy for creative and cultural education is essential to that process. We put the case for developing creative and cultural education; we consider what is involved; we look at current provision and assess the opportunities and obstacles; and we set out a national strategy. By creative education we mean forms of education that develop young peopleʻs capacities for original ideas and action: by cultural education we mean forms of education that enable them to engage positively with the growing complexity and diversity of social values and ways of life. We argue that there are important relationships between creative and cultural education, and significant implications for methods of teaching and assessment, the balance of the school curriculum and for partnerships between schools and the wider world.

In modern educational theory, creativity, as the ability to build a unique product, create new, unique solutions to complex problems and approaches to challenging tasks, is a students’ priority competence (Rotherham and Willingham, 2010; Donovan et al., 2014). Based on the results of a review of modern publications on creativity in education, we identify a number of research areas, which include the study of trends in the development of creative potential, creative abilities and cognitive styles; environmental conditions that promote or hinder creativity; links between creativity and learning models; development of techniques teaching creativity (creative learning’, teaching for creativity) and technologies aiming to increase creativity and unlock creative potential (Runco, 2007; Newton and Beverton, 2012; Newton and Newton, 2014; Gruszka and Tang, 2017; Runco et al., 2020). The most important factors that determine the child’s creativity (Lebuda et al., 2021) are creative potential and creative abilities (Kim, 2005), general cognitive abilities (Zabelina and Ganis, 2018; Gerwig et al., 2021), specific skills in a particular subject area (Simonton, 2009; Szen-Ziemiańska et al., 2017; Ahmed and Feist, 2021); learning (Kaufman and Kaufman, 2007; Agoguéa et al., 2014) in an enriched cultural and educational environment (Vygotsky, 2001).

At the same time, experts in the field of modern education and educational policy are faced with a certain kind of contradiction. On the one hand, the research highlights the important role of education in encouraging and developing children’s creativity (Thurlings et al., 2015). On the other hand, due to diversification (variability of educational services and educational curricula, types and kinds of educational institutions, teaching methods and techniques) and standardized testing of basic skills, children’s creativity actually decreases as they move along their educational trajectory (Robinson, 2011; Kupers et al., 2019).

We believe that by finding answers to our research questions we will be able to resolve the identified contradictions.

In our research, Vygotsky’s theory is implemented by means of fundamentally important theoretical provisions:

1. A creative cultural and educational environment, as an accumulator of psychological tools, is the source of the child’s mental and personal development.
2. Higher mental functions, as a result of the internalization of psychological tools, are formed in learning by assimilating historically developed methods and forms of activity, both as a way of the student’s interaction with the educational environment, and as a form of the student’s cooperation with others.
3. In order to create a zone of proximal development and to give rise to a number of internal development processes, we need a properly constructed school education and a properly organized educational environment.

### 1.2. Aims and objectives of the research

The purpose of our research is to study the creative potential as psychological capacities for younger schoolchildren’s creative self-realization and self-development in various conditions of the educational environment.

### 1.3. Research objectives

1. Conduct a comprehensive expert assessment of the educational environment qualitative parameters, identifying four types of schools with different severity of characteristics: serene, dogmatic, career and creative.
2. Identify indicators of younger schoolchildren’s creative potential and personal qualities in accordance with the variable parameters of the educational environment.

## 2. Materials and methods

The methodological basis of this work is the conceptual provisions of Vygotsky’s cultural-historical psychology. We distinguish both external determinants (a specially organized educational environment) and internal factors, whose actions explain such phenomena as the zones of actual, proximal and further development.

### 2.1. Schools and participants

Our study of junior schoolchildren’s creative potential was conducted on the basis of Kazan state schools corresponding to various pedagogical models of organizing education (a gymnasium with in-depth study of individual subjects - English, biology, mathematics and physics; “Specialized Olympiad and Scientific Center ‘Sun’,” a general education boarding school; two schools with a general education curricula).

Nine experts assessed the school educational environment using Yasvin’s method of vector modeling: psychologists and teachers of educational institutions, university professors and master students of Kazan. All diagnostic procedures were carried out in full accordance with the diagnostic standard: using uniform forms, instructions and stimulus materials. The reliability of the study results was ensured by the preliminary training of experts in a series of workshops that were devoted to the development of a consensus assessment. We revealed a high degree of consistency in observations found in the experts’ assessments.

Our empirical study included 160 4th grade students without developmental delays or disabilities, aged 9–10 years (*n* = 160, *M* = 9.5 years, SD = 2.6; 49% boys), with written parental consent; among them 40 children were from the gymnasium with in-depth study of individual subjects - English, biology, mathematics and physics (17 boys, 23 girls), their parents’ education: 78% - higher, 22% - secondary vocational, the family social status: 45% - workers, 15% - engineers, employees, 40% - entrepreneurs, businessmen; 40 children were from the general education boarding school “Specialized Olympiad-Scientific Center ‘Sun’” (21 boys, 19 girls), their parents’ education: 83% - higher, 17% - specialized secondary; the family social status: 70% - workers, 13% - engineers, employees, 17% - entrepreneurs, businessmen; 80 children studied according to the general education curriculum (40 boys, 40 girls), their parents’ education: 58% - higher, 42% - specialized secondary; the family social status: 87% - workers, 3% - engineers, employees, 10% - entrepreneurs, businessmen.

### 2.2. Measures

#### 2.2.1. Assessment of the schools’ educational environment

To study the features of the educational environment, Yasvin identifies 11 parameters (five ‘main’ characteristics: breadth, intensity, modality, degree of awareness and stability, and six ‘secondary’ characteristics: emotionality, generality, dominance, coherence, mobility and agency). This method is characterized by the construction of a vector that corresponds to a certain type of educational environment. This operation is carried out after counting up the answers to diagnostic questions: three of them aim to determine the opportunities for the student’s free development in the educational environment, and three more show the availability of opportunities for the development of the child’s agency. Further, in the coordinate system (agency-inaction, freedom-dependence), a vector is built showing the type of environment, which constitutes modality as a feature of the educational environment.

Diagnostic questions and interpretation of the answers.

**For the “freedom-dependency” axis:**

1. Whose interests and values come first in this educational environment?

(a) personality; (b) society (group).

The priority of personal interests and values over the interests and values of society is interpreted as an opportunity for free development, and a score is accordingly marked on the “freedom” scale; in case of the priority of public interests, a score is marked on the scale “dependence.”

2. Who usually adjusts to whom in the process of interaction?

(a) the teacher to the students; (b) the students to the teacher.

If it is noted that in the given educational environment, the situation when the teacher adjusts to the students (or at least the teacher strives for this situation) dominates, this is interpreted as an opportunity for the students’ free development, respectively, a score is marked on the “freedom” scale; if it is stated that students are constantly forced to obey their teachers, a score is marked on the “dependence” scale.

3. What form of education is predominantly carried out in this educational environment?

(a) individual; (b) collective (team).

The educational environment with individual-oriented forms of learning is interpreted as the environment possessing additional opportunities for the free development of a self-directed student, and a score is given on the “freedom” scale; in the case when teamwork has priority in the educational environment, a score is marked on the “dependency” scale.

**For the “Agency –Inaction” axis:**

4. Is punishment of the child practiced in this educational environment?

(a) yes; (b) no.

The absence of punishment is considered as a condition conducive to the development of agency; thus, a score is given on the “agency” scale; in the case when punishments are practiced (both directly and indirectly) in this learning environment, a score is given on the “inaction” scale.

5. Does the given educational environment stimulate the manifestation of any children’s initiative?

(a) more often yes; (b) usually not.

If in this learning environment, positive reinforcement of student initiatives is observed, then this is interpreted as an additional opportunity for the development of students’ agency and a score is given on the “agency” scale; if the initiative demonstrated by the child is usually ignored or can lead to all sorts of troubles, then a score is marked on the “inaction” scale.

6. Do certain children’s creative manifestations find any positive response in this educational environment?

(a) more often yes; (b) usually not.

In the case when the learning environment encourages or appreciates creativity, such an environment is considered as conducive to the development of agency, a score is marked on the “agency” scale; if the children’s creative self-expression is ignored and goes unnoticed and underestimated, a score is marked on the “inaction” scale.

**Figure 1:**
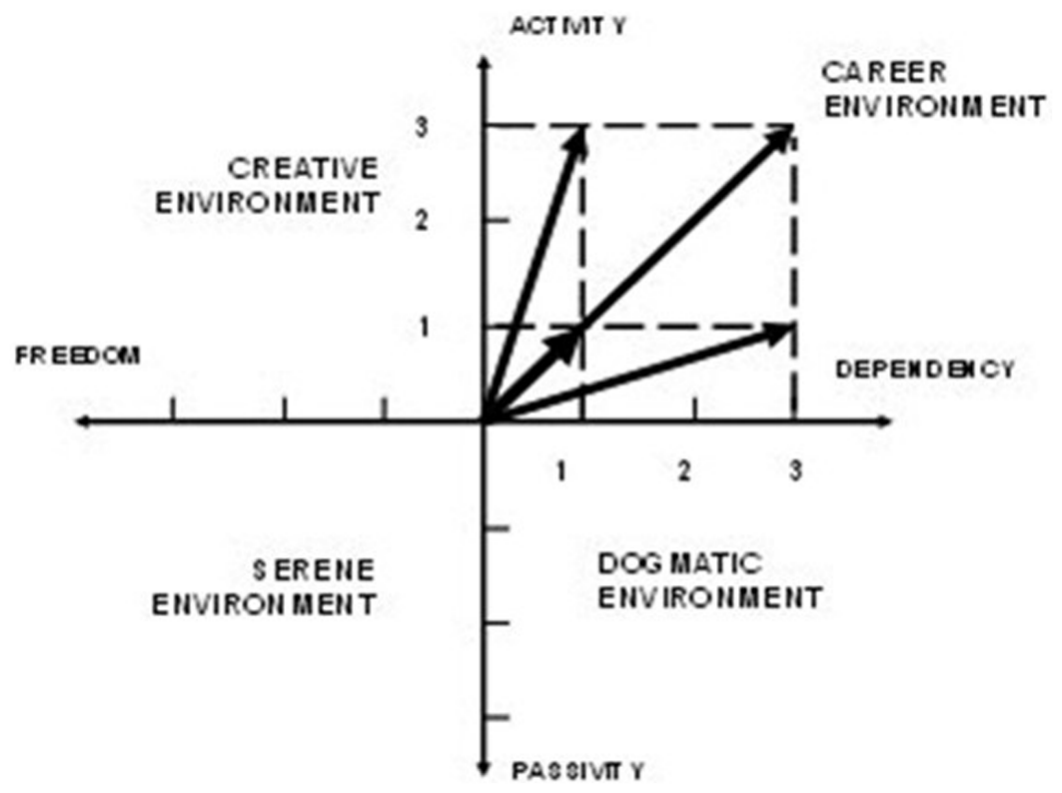
An example of the possible construction options of a vector model of the environment based on the answers to diagnostic questions. Reproduced from Yasvin et al. (2015), licenced under CC BY 4.0.

The author proposes four basic types of educational environment: “dogmatic” (contributes to the development of passive behavior and dependence of the child); “career” (contributes to the development of agency and the dependence of the child at the same time); “serene” (promotes the free development, but causes the formation of the child’s passive behavior); “creative” (contributes to the free development of an active child). Based on the answers to the diagnostic questions, corresponding vector, which allows one to assess the learning environment, is constructed in the coordinate system (Figure 1); an example of the possible construction options of a vector model of the environment based on the answer to diagnostic questions). The studies of Yasvin provide a detailed description of the methodology for examining a school educational environment and the typology of educational environments at schools (Yasvin et al., 2015; Yasvin, 2020).

#### 2.2.2. Creative potential

We used a test of verbal creativity when studying the creative potential of younger schoolchildren. It includes two qualitative characteristics: “originality index” and “uniqueness index.” The technique aims to identify and assess the teste’s often hidden, blocked creative potential. The proposed method is a Russian-language adapted version of the RAT test (remote association test) by Mednik (2006). The Remote Associates Test (RAT) is a creativity test used to determine a human’s creative potential. The test typically lasts 40 min and consists of thirty to forty questions each of which consists of three common stimulus words that appear to be unrelated. The subject must think of a fourth word that is somehow related to each of the first three words. Scores are calculated based on the number of correct questions.^1^ The technique was adapted by Alekseeva and Galkina in the Druzhinin’s Laboratory of the Abilities Psychology at the Institute of Psychology, the Russian Academy of Sciences, based on a sample of schoolchildren; Voronin based his study on a sample of managers aged 23 to 35 years. For the Russian version Cronbach’s coefficient is α = 0.87 (Ushakov, 2011; Druzhinin, 2019).

The Johnson Creativity Inventory was used as adapted by Tunik (1997a, 1998), based on two approaches:

- according to Torrens, creativity manifests itself with a lack of knowledge; in the process of incorporating information into new structures and relationships; in the process of identifying missing information; in the process of finding new solutions and testing them; in the process of reporting results;
- according to Johnson (1979), creativity manifests itself as an unexpected productive act performed spontaneously by the performer in a certain environment of social interaction. In this case, the performer relies on his/her own knowledge and capabilities.

This creativity questionnaire focuses on the elements that are associated with creative self-expression. The Creativity Inventory is an objective, eight-item checklist of creative thinking and behavior characteristics, designed specifically to identify externally observable manifestations of creativity.

Each statement of the questionnaire is evaluated on a scale containing five gradations (possible rating points: 1 - never, 2 - rarely, 3 - sometimes, 4 - often, 5 - always). The overall creativity score is the sum of scores for eight items (the minimum score - 8, the maximum score - 40 points). The Table 1 shows the correspondence of the sum of points to the levels of creativity. The internal consistency of the Russian version of the scale was Cronbach’s alpha α = 0.79. To assess the retest reliability, the correlation coefficient of Spearman ranks was calculated (interval - three months), which turned out to be 0.78 (sample size - 80 children). To compare the data of various experts (the experts were three teachers teaching different subjects), Spearman’s correlation coefficients were found. For a sample of 8-year-old children, the value of the correlation coefficient ranged from 0.51 to 0.71, for a sample of 10-year-old children - from 0.49 to 0.78, for a sample of 14-year-old children - from 0.58 to 0.79. It should also be noted that with the increase in the age of children, the consistency of the data of various experts among themselves increases (Tunik, 1997b, 1998, 2000).

**Table 1:** Levels of Creativity adapted from Tunik (1997b).

Creativity level	Sum of points
Very high	40–34
High	33–27
Medium	26–20
Low	19–15
Very low	14–8

To study the level of communicative control, the test “Diagnostics of communicative control” by Schneider was used (Schneider, 2002). The test consists of 10 statements reflecting reactions to some communication situations. The internal consistency of the Russian version of the test is α = 0.85.

To assess the personal qualities of younger students, we used a modified version of the children’s personality questionnaire intended for 8-12 year-old children and developed by Cattell and Koan (Children Personality Questionnaire – CPQ). The internal consistency of the Russian version of the test is α = 0.88 (Alexandrovskaya and Gilyashev, 1978, 1995).

### 2.3. Research results

**Figure 2:**
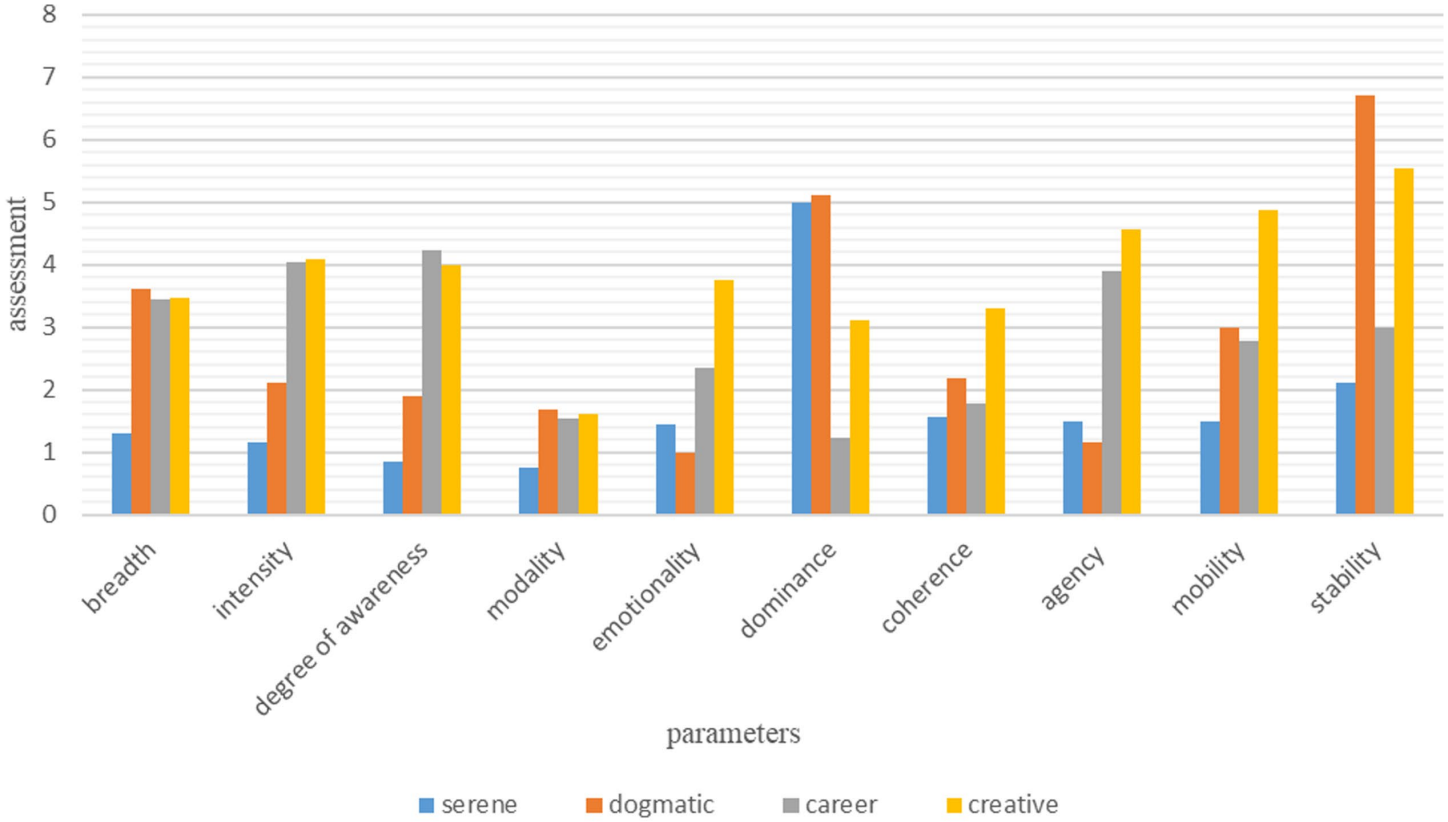
Severity of the essential characteristics of educational environments based on vector modeling (V. Yasvin).

In the course of solving the first task, we performed a comprehensive expert assessment of the qualitative parameters of the educational environment based on the parameters formulated by Yasvin (2020). As a result, we identified four types of schools with different severity degrees of the essential characteristics of educational environments: serene, dogmatic, career and creative (see Figure 2). In the course of the study, we found that the general education spaces of Kazan schools are more consistent with the dogmatic and serene environment, a career type of modality characterizes one of the gymnasiums, and the “Specialized Olympiad and Scientific Center ‘Sun’ has a creative development environment. The histogram shows that dogmatic schools are characterized by high stability and the ability to quickly adapt to external pressure, by a clear internal organization of the system, respect for traditions and order. However, it should be noted that this educational environment shows low agency and emotionality, demonstrated by the subjects of educational relations.

As can be seen from the histogram, a serene-type general education school is characterized by high dominance, the significance of this local environment in the system of values of the subjects of the educational process. A distinctive feature of a serene environment is relatively low stability, manifested in the precariousness of its system, low rates of awareness, intensity, generalization and mobility of the educational process. “There is no perseverance either in the desire to preserve, hold out, or in the desire to achieve, find. The child lives in an atmosphere of internal well-being and lazy, conservative habits, condescension to modern trends, among attractive simplicity” (Korchak, 1980).

According to the data obtained on a multidisciplinary career-type gymnasium, we have found that the features of this environment are characterized by a high level of intensity and awareness due to a deeper study of individual subjects and the focus of all activities on achieving the set goals. This environment shows high agency, which indicates the ability to produce socially significant results with a beneficial impact on society.

Specialized Olympiad and Scientific Center ‘Sun’ is distinguished by a creative environment with cultural content based on ethno-cultural samples and norms with bright national color, mobility and emotional richness of the educational process. Teachers have the ability to creatively approach the organization of the educational process, namely, to use new methodological developments, to conduct lessons in the context of certain events taking place in the environment; they vary the lesson plan depending on the specific situation, get acquainted with the work of psychologists and, accordingly, restructure the nature of their pedagogical communication with their students, etc.

While solving the second empirical problem, we subjected the obtained empirical data to a one-way analysis of variance (see Tables 2, 3). Data processing methods were carried out using the IBM SPSS Statistics 23 for Windows statistical package: descriptive statistics and the analysis of variance (univariate one-way ANOVA). The Scheffe *a posteriori* method of paired comparisons (Scheffe test) made it possible to carry out pairwise multiple comparisons of mean values while obtaining a statistically significant result.

**Table 2:** Mean values and standard deviations for indicators of younger schoolchildren’s creative potential in terms of variability of educational environments (univariate one-way ANOVA).

Creative potential indicators	Types of educational environments based on vector modeling (V. Yasvin)			
	serene	dogmatic	career	creative
Originality index (The Remote Associates Test (RAT)	0.73 (0.85)	0.76 (0.87)	0.65*(0.81)	0.82*(0.91)
Uniqueness index (RAT)	0.58 (0.30)	0.58*(0.14)	0.51*(0.19)	0.74*(0.08)
Creativity (The Johnson Creativity Inventory)	24.00 (2.35)	23.00 (6.10)	26.00 (3.52)	25.00 (5.62)
Communicative control (Schneider)	5.77 (1.09)	5.20 (1.42)	6.47 (1.29)	5.65 (1.98)

The main effects of the “types of educational environments” factor: originality index *F* (2, 69) = **12.03**, *p* ≤ 0.01; uniqueness index *F* (2, 69) = **12.15**, *p* ≤ 0.01; creativity *F* (2, 69) = 2.07, *p* ≥ 0.05; Communicative control *F* (2, 69) = 2.60, *p* ≥ 0.05 (significant differences are given in bold). An asterisk (*) marks the groups that significantly differ from each other in terms of the Scheffe correction results.

**Table 3:** Mean values and standard deviations for indicators of younger schoolchildren’s creative potential in terms of variability of educational environments (univariate one-way ANOVA).

Indicators of personal characteristics (Children Personality Questionnaire – CPQ)	Types of educational environments based on vector modeling (V. Yasvin)			
	Serene	Dogmatic	Career	Creative
Sociability	4.80 (0.83)	5.00 (2.03)	3.77 (1.62)	5.17 (2.59)
Verbal intelligence	5.40* (2.30)	3.69* (1.88)	7.49* (1.48)	7.41* (1.65)
Self-confidence	4.60* (1.67)	7.46* (1.66)	4.56* (1.69)	5.17* (2.05)
Excitability	3.40* (1.14)	6.38* (1.75)	7.84* (1.37)	5.51* (2.13)
Tendency for self-affirmation	5.20* (2.48)	6.07* (1.25)	7.73* (1.28)	4.75* (2.06)
Propensity to take risks	4.80* (1,09)	5.07 (1.03)	6.00* (1.88)	7.15* (1.75)
Responsibility	6.00 (0.70)	5.00 (2.00)	4.60 (1.79)	5.58 (2.22)
Social courage	4.80 (2,38)	4.00* (1.77)	4.62 (2.36)	7.75* (1.55)
Sensitivity	8.00* (1,22)	5.07* (1,89)	3.56* (1.9)	5.72* (2.51)
Anxiety	4.20* (1,09)	7.61* (1.44)	7.05* (1.94)	3.68* (1.81)
Self-control	4.80 (1,48)	4.46 (1.80)	3,54 (2.01)	5.06 (2.08)
Nervous tension	4.00* (0.83)	7.00* (1.44)	7.00* (1.74)	4.00* (1.25)

The main effects of the “types of educational environments” factor: sociability *F* (2, 69) = 1.68, *p* ≥ 0.05; verbal intelligence *F* (2, 69) = **21.36**, *p* ≤ 0.01; self-confidence *F* (2, 69) = **9.13**, *p* ≤ 0.01; excitability *F* (2, 69) = **19.78,** *p* ≤ 0.01; tendency for self-assertion *F* (2, 69) = **23.04**, *p* ≤ 0.01; propensity to take risks *F* (2, 69) = **5.68**, *p* ≤ 0.01; responsibility *F* (2, 69) = 2.09, *p* ≥ 0.05; social courage *F* (2, 69) = **16.84**, *p* ≤ 0.01; sensitivity *F* (2, 69) = **11.85**, *p* ≤ 0.01; anxiety *F* (2, 69) = **26.49**, *p* ≤ 0.01; self-control *F* (2, 69) = 2.35, *p* ≥ 0.05; nervous tension *F* (2, 69) = **33.16**, *p* ≤ 0.01 (significant differences are given in bold). An asterisk (*) marks the groups that differ significantly from each other in terms of the Scheffe correction results.

Table 1 illustrates the data on the severity of the creative potential of the schoolchildren (see Table 1), who received higher scores according to the “Originality index” (the ability to express themselves in unusual activities and situations), and the “Uniqueness index” (the ability to make unconventional judgments and perform unusual actions) from the creative educational environment.

As can be seen in Table 2, there are statistically confirmed differences in the manifestation of such qualities as verbal intelligence, self-confidence, a tendency for self-affirmation, propensity to take risks, social courage, sensitivity, excitability, anxiety and nervous tension in younger schoolchildren from different educational environments.

We have established that younger schoolchildren from **creative and career environments** have the highest rates of verbal intelligence. They master new knowledge and develop abstract thinking faster. Schoolchildren from the **creative environment** are characterized by risk-taking and high social courage to a greater degree. These children are distinguished by dynamism and agency; when faced with non-standard situations, they do not get lost and quickly find a different way to solve the problems that have arisen. Moreover, we recorded low levels of anxiety and nervous tension, which ensures emotional stability in educational and cognitive activities. **Career-type** schoolchildren have a tendency to self-affirmation, a desire for leadership and dominance with excessive motivation, practicality and realism in resolving problem situations. They are characterized by low sensitivity, increased excitability and nervous tension with the need for practical relaxation in the process of activity. The characteristic features of younger schoolchildren from a dogmatic environment are: being better prepared to successfully meet school requirements, however demonstrating low social courage. These schoolchildren more often use standard approaches to solving problems, producing elementary forms of thinking, which is accompanied by variability in mood and a change in mental states. The younger schoolchildren from a **serene environment** are largely exposed to the influences of the external environment, they are distinguished by the absence of strong motives and intentions in achieving goals, low rates of risky behavior combined with a need for support from others.

## 3. Conclusion

The paper analyzes various types of educational environments in terms of culturally-appropriate components. Based on the results of the dispersion analysis, the study revealed younger schoolchildren’s creative potential in the context of educational environment variability, and the relationships between the parameters of the educational environment and the personal characteristics of children.

Thus, in a creative educational environment, to a greater extent than in other environments, the subjects of the educational system provide and actively support the individual development of the child and the disclosure of its creative potential. The priority of the creative educational environment is not only to develop the child’s agency and creativity, but also to boost its own need for creativity and self-development as the creation of the self and the formation of the ability to independently set goals and realize its own ideas. The discovered empirical regularity is not a heuristic one in science, it is an independent trend in pedagogical practices. Pedagogical intervention is aimed not so much at meeting the requirements of the teacher, but rather at satisfying the need for creativity by involving schoolchildren in mental, intellectual and communicative activities.

Our study statistically confirmed significant differences between educational environments in terms of younger schoolchildren’s creative potential. Thus, in a *creative educational environment*, younger schoolchildren demonstrate higher subjective activity with the possibility of unique achievements in educational and cognitive sphere, the desire for unusual actions and unconventional judgments due to verbal intelligence. In a *career educational environment*, learners are more prone to self-affirmation, the desire for leadership and dominance. In a *dogmatic educational environment*, schoolchildren most often use standard approaches to solving assigned problems, generating elementary forms of thinking. In a *serene educational environment*, schoolchildren do not have strong motivations and intentions to achieve goals, risk-taking behavior is not typical, and they demonstrate a high need for support from the outside world.

Thus, pedagogical conditions, as components of the educational system, reflect the totality of the educational environment possibilities expressed in the capabilities of the educational process subjects. Vygotsky’s ideas not only complement modern ideas about the relationship between learning and the psyche development, but also reveal the problems of the experimental evidence base in other modern approaches. This, in particular, concerns the clarification of the mechanisms in the relationship between learning and mental development in the context of controlled initiation from the outside of the self-organization processes of the cognitive system elements in the subject of education in accordance with the system self-development potential (Pogozhina, 2016).

**The limitations of the study** apply to the choice of: (1) the subject of the study, in particular, the creative potential was studied in connection with the parameters of the educational environment in different types of primary schools; the effects of external and internal factors in determining the creative potential were not considered; (2) strategies for building groups (arranging a sample) involving 160 junior schoolchildren; it is necessary to increase the sample to prevent internal threats to the validity of the study.

**Research prospects** concern the clarification of the mechanisms of the relationship between learning and the mental development of schoolchildren of different ages in the context of controlled initiation from the outside of the processes of self-organization of elements of creativity in accordance with the potential for self-development of a complex self-organizing system of higher mental functions (Vygotsky, 1999, 2005).

**Practical value.** The results of the study can be used by specialists in the design and evaluation of educational environments; by school psychologists, working with younger schoolchildren in the course of implementation of differentiated, individual approaches in the education system.

## Data availability statement

The original contributions presented in the study are included in the article/Supplementary material, further inquiries can be directed to the corresponding author.

## Ethics statement

The studies involving human participants were reviewed and approved by the Committee of the Institute of Psychology and Education of Kazan (Volga Region) Federal University. Written informed consent to participate in this study was provided by the participants’ legal guardian/next of kin.

## Author contributions

VK designed and directed the project, and developed the theoretical framework. ES performed the research and conducted a mathematical analysis of the data, performed the analysis, drafted the manuscript, and aided in interpreting the results and worked on the manuscript. All authors discussed the results and contributed to the final manuscript.

## Funding

This work was supported by the Kazan Federal University Strategic Academic Leadership Program (PRIORITY-2030).

## Conflict of interest

The authors declare that the research was conducted in the absence of any commercial or financial relationships that could be construed as a potential conflict of interest.

## Publisher’s note

All claims expressed in this article are solely those of the authors and do not necessarily represent those of their affiliated organizations, or those of the publisher, the editors and the reviewers. Any product that may be evaluated in this article, or claim that may be made by its manufacturer, is not guaranteed or endorsed by the publisher.

## Footnotes

^1^RAT https://en.wikipedia.org/wiki/Remote_Associates_Test.

## References

[ref1] Agoguéa M. Poirel N. Pineau A. Houdé O. Cassotti M. (2014). The impact of age and training on creativity: a design-theory approach to study fixation effects. Think. Skills Creat. 11, 33–41. doi: 10.1016/j.tsc.2013.10.002

[ref2] Ahmed S. T. Feist G. J. (2021). The language of creativity: validating linguistic analysis to assess creative scientists and artists. Front. Psychol. 12:724083. doi: 10.3389/fpsyg.2021.724083, PMID: 34867602 PMC8639503

[ref3] Alexandrovskaya E. M. Gilyashev I. N. (1995). An adapted modified version of R. Cattell’s children’s personality questionnaire: methodological recommendations. Psychodiagnostics of children and adolescents. Moscow: Folium.

[ref1002] Aleksandrovskaya E. M. Gilyaweva I. N. (1978). Verification of the adapted personality questionnaire R.Cattella (CPQ). Psychological research methods of personality in the clinic. Leningrad, 70–76., PMID:

[ref4] Donovan L. Green T. Mason C. (2014). Examining the 21st century classroom: developing an innovation configuration map. J. Educ. Comput. Res. 50, 161–178. doi: 10.2190/EC.50.2.a

[ref5] Druzhinin V.N. (2019). Psychology of general abilities: a textbook for universities. (3rd Edn). Moscow: Yurayt Publishing House.

[ref6] Gerwig A. Miroshnik K. Forthmann B. Benedek M. Karwowski M. Holling H. (2021). The relationship between intelligence and divergent thinking – a meta-analytic update. J. Intelligence 9:23. doi: 10.3390/jintelligence9020023, PMID: 33923940 PMC8167550

[ref7] Gruszka A. Tang M. (2017). “"the 4P’s creativity model and its application in different fields," world scientific book chapters” in Нandbook of the management of creativity and innovation: theory and practice. eds. Tang M. Werner C. H. (Singapore: World Scientific Publishing Co. Pte. Ltd), 51–71.

[ref8] Johnson D. L. (1979). Creativity Checklist. Educational Resources Information Center (ERIC).

[ref9] Kaufman S. B. Kaufman J. C. (2007). Ten years to expertise, many more to greatness: an investigation of modern writers. J. Creat. Behav. 41, 114–124. doi: 10.1002/j.2162-6057.2007.tb01284.x

[ref10] Khotinets V. Y. Medvedeva D. S. (2021). Peculiarities of verbal and mental activity of children of monolinguals and natural bilinguals. Psikhologicheskiy zhurnal 42, 25–35. doi: 10.31857/S020595920014236-5

[ref11] Khotinets V. Shishova E. Zinnurova E. Kozhevnikova O. Medvedeva D. Novgorodova Y.. (2022). The development of cognitive regulation in connection with the communicative competence of monolingual and balanced bilingual children. Educ. Self Dev. 17, 317–334. doi: 10.26907/esd.17.3.22

[ref12] Kim K. H. (2005). Can only intelligent people be creative? A meta-analysis. J. Second. Gift. Educ. 16, 57–66. doi: 10.4219/jsge-2005-473

[ref13] Korchak Ya. (1980). How to love a child. Publishing house: Kniga.

[ref14] Kupers E. Lehmann-Wermser A. McPherson G. van Geert P. (2019). Children's creativity: a theoretical framework and systematic review. Rev. Educ. Res. 89, 93–124. doi: 10.3102/0034654318815707

[ref15] Lebuda I. Zielińska A. Karwowski M. (2021). On surface and core predictors of real-life creativity. Think. Skills Creat. 42:100973. doi: 10.1016/j.tsc.2021.100973

[ref16] Leontyev A.N. (1975). Activity, consciousness, personality. Politizdat, Мoscow.

[ref17] Test of verbal creativity of Mednik S. (2006). Collection of psychological tests. Part II: a manual. Comp. E. E. Mironova. Minsk: ENVILA.

[ref18] Newton L. Beverton S. (2012). Pre-service teachers’ conceptions of creativity in elementary school English. Think. Skills Creat. 7, 165–176. doi: 10.1016/j.tsc.2012.02.002

[ref19] Newton L. Newton D. (2014). Creativity in 21st-century education. Prospects 44, 575–589. doi: 10.1007/s11125-014-9322-1

[ref20] Pogozhina I. N. (2016). The models of relationship between training and psyche development in cultural-historical and activity approaches. Psychol. Sci. Educ. 8:16. doi: 10.17759/psyedu.2016080302

[ref21] Robinson K. (2011). Out of our minds. Chichester, England: Capstone.

[ref22] Rotherham A. J. Willingham D. T. (2010). 21st-century skills: not new, but a worthy challenge. American educator, Spring, 17–20. Available at: http://www.aft.org/sites/default/files/periodicals/RotherhamWillingham.pdf

[ref23] Rubtsov V. V. Ulanovskaya I. M. (2022). The influence of ways of organizing learning interactions on the development of communicative and reflexive abilities of children 6–10 years old. Psikhologicheskaya nauka i obrazovanie 27, 5–6. doi: 10.17759/pse.2022270101

[ref24] Runco M. A. (2007). A hierarchical framework for the study of creativity. New Horizons in Education 55, 1–9. Available at: https://files.eric.ed.gov/fulltext/EJ832891.pdf

[ref25] Runco M. A. Acar S. (2012). Divergent thinking as an Indicator of creative potential. Creat. Res. J. 24, 66–75. doi: 10.1080/10400419.2012.652929

[ref26] Runco Mark Pritzker Steven Reiter-Palmon Roni, “Encyclopedia of creativity, 3rd Edition” (2020). Psychology Faculty Books and Monographs. 7.

[ref27] Schneider M. (2002). “Diagnosis of communicative control” in Socio-psychological diagnostics of personality development and small groups. eds. Fetiskin N. P. Kozlov V. V. Manuilov G. M. (Moscow, Russia: Publishing House of the Institute of Psychotherapy).

[ref28] Shishova E. O. Akhatova A. Z. (2022). Interaction of communicative creativity and primary school Students' creativeness in the context of educational environment. New Ideas Child Educ Psychol 2, 66–79. doi: 10.11621/nicep.2022.0204

[ref29] Simonton D. K. (1995). Exceptional personal influence: an integrative paradigm. Creat. Res. J. 8, 371–376. doi: 10.1207/s15326934crj0804_3

[ref30] Simonton D. K. (2009). Varieties of (scientific) creativity: a hierarchical model of domain-specific disposition, development, and achievement. Perspect. Psychol. Sci. 4, 441–452. doi: 10.1111/j.1745-6924.2009.01152.x, PMID: 26162214

[ref31] Szen-Ziemiańska J. Lebuda I. Karwowski M. (2017). “Mix and match: opportunities, conditions, and limitations of cross-domain creativity” in The Cambridge handbook of creativity across domains. eds. Kaufman J. C. Glăveanu V. P. Baer J. (Cambridge: Cambridge University Press), 18–40.

[ref32] The National Advisory Committee on Creative and Cultural Education (NACCCE). (1999). All our futures: creativity, culture and education, department for education and employment. London: DFEE.

[ref33] Thurlings M. Evers A. T. Vermeulen M. (2015). Toward a model of explaining teachers’ innovative behavior: a literature review. Rev. Educ. Res. 85, 430–471. doi: 10.3102/0034654314557949

[ref34] Tunik E. E. (1997a). Johnson’s creativity questionnaire. St. Petersburg: SPbUPM.

[ref1004] Tunik E. E. (1997b). Psychodiagnostics of creative thinking. Creative tests. SPb.: SPbUPM.

[ref35] Tunik E. E. (1998). Diagnostics of creativity. E. Torrens test. St. Petersburg: Imaton.

[ref9001] Tunik E. E. (2000). Johnson creativity questionnaire. Sch. Psychol. 47, 8–9.

[ref1003] Ushakov D. V. (2011). Psychology of intelligence and giftedness. Moscow: Institute of Psychology, Russian Academy of Education, Academy of Sciences.

[ref36] Veraksa N. E. (1990). Dialectical thinking and creativity. Ques. Psychol. 4, 5–9.

[ref37] Veraksa N. E. (2018). Child development: two paradigms. Cult.-Hist. Psychol. 14, 102–108. doi: 10.17759/chp.2018140211

[ref38] Veraksa A. Gavrilova M. Bukhalenkova D. (2019). Relationship between the procedural quality of the educational environment and speech development indicators. Ques. Educ. 2, 159–178. doi: 10.17323/1814-9545-2019-2-159-178

[ref39] Vygotsky L. S. (1935). “Education and development during school age” in Report to the National Vygotsky's works conference of preschool education. In mental development of children during education (Moscow-Leningrad: Uchpedgiz), 20–32.

[ref40] Vygotsky L. S. (1984). “The problem of age // Vygotsky L.S. collected works” in 6 volumes: 4 (Moscow: Pedagogy).

[ref41] Vygotsky L. S. (1999). Questions of child psychology. St. Petersburg: Peter.

[ref42] Vygotsky L.S. (1999). Thinking and speech. Moscow: Labyrinth.

[ref43] Vygotsky L.S. (2001). Lecture on Pedology. Izhevsk: Udmurt University Publishing House.

[ref44] Vygotsky L. S. (2004). Imagination and creativity in childhood. J. Russ. East Eur. Psychol. 42, 7–97. doi: 10.1080/10610405.2004.11059210

[ref45] Vygotsky L.S. (2005). Psychology of human development. Moscow: Eksmo-Press, Smysl.

[ref47] Yasvin V.A. (2010). School as a developing environment: Monograph. M.: Institute of Scientific Information and Monitoring of the Russian academy of education. (Series: scientific and publishing project in support of the national educational initiative "Our New School").

[ref48] Yasvin V.A. (2020). Instrumental expertise in the process of Pedagogical design of the School Environment: author’s abstract of doctoral dissertation. Moscow: Moscow City Pedagogical University (In Russ.).

[ref49] Yasvin V. A. Rusetskaya M. N. Osadchiy M. A. (2015). Assessment of school and university environments by high school and college students. Biomed. Pharmacol. J. 8, 761–772. doi: 10.13005/bpj/824

[ref50] Zabelina D. L. Ganis G. (2018). Creativity and cognitive control: behavioral and ERP evidence that divergent thinking, but not real-life creative achievement, relates to better cognitive control. Neuropsychologia 118, 20–28. doi: 10.1016/j.neuropsychologia.2018.02.014, PMID: 29447843

