# Supplementary material for: Cultural and educational environment in the development of younger schoolchildren’s creative potential
Source: Front Psychol. 2023 Sep 15;14:1178535. doi: 10.3389/fpsyg.2023.1178535 (PMC10541215; doi:10.3389/fpsyg.2023.1178535)
Supplement: Supplementary file 1 [file Data_Sheet_1.docx]

Supplementary Material

- **Cultural and Educational Environment in the Development of Younger Schoolchildren's Creative Potential**

**Vera Yu. Khotinets, Evgeniya O. Shishova^*^**

*** Correspondence:**Evgeniya O. Shishova
Evgeniya.Shishova@kpfu.ru

# Supplementary Tables

**Supplementary Table 1.** Mean values and standard deviations for indicators of younger schoolchildren’s creative potential in terms of variability of educational environments (univariate one-way ANOVA)

| Creative potential indicators | Types of educational environments based on vector modeling  (V. Yasvin) | | | |
| --- | --- | --- | --- | --- |
|  | serene | dogmatic | career | creative |
| Originality index (The Remote Associates Test (RAT) | 0,73 (0,85) | 0,76 (0,87) | 0,65*(0,81) | 0,82*(0,91) |
| Uniqueness index (RAT) | 0,58 (0,30) | 0,58*(0,14) | 0,51*(0,19) | 0,74*(0,08) |
| Creativity (The Johnson Creativity Inventory) | 24,00 (2,35) | 23,00 (6,10) | 26,00 (3,52) | 25,00 (5,62) |
| Communicative control (Schneider) | 5,77 (1,09) | 5,20 (1,42) | 6,47 (1,29) | 5,65 (1,98) |

**Note.** The main effects of the “types of educational environments” factor: originality index F (2, 69) = **12.03**, p ≤ 0.01; uniqueness index F (2, 69) = **12.15**, p ≤ 0.01; creativity F (2, 69) = 2.07, p ≥ 0.05; communicative control F (2, 69) = 2.60, p ≥ 0.05 (significant differences are given in bold). An asterisk (*) marks the groups that significantly differ from each other in terms of the Scheffe correction results.

**Supplementary Table 2.** Mean values and standard deviations for indicators of younger schoolchildren’s creative potential in terms of variability of educational environments (univariate one-way ANOVA)

| Indicators of personal characteristics  (Children Personality Questionnaire – CPQ) | Types of educational environments based on vector modeling (V. Yasvin) | | | |
| --- | --- | --- | --- | --- |
|  | serene | dogmatic | career | creative |
| Sociability | 4,80 (0,83) | 5,00 (2,03) | 3,77 (1,62) | 5,17 (2,59) |
| Verbal intelligence | 5,40*(2,30) | 3,69*(1,88) | 7,49*(1,48) | 7,41* (1,65) |
| Self-confidence | 4,60*(1,67) | 7,46*(1,66) | 4,56*(1,69) | 5,17*(2,05) |
| Excitability | 3,40*(1,14) | 6,38*(1,75) | 7,84*(1,37) | 5,51*(2,13) |
| Tendency for self-affirmation | 5,20*(2,48) | 6,07*(1,25) | 7,73*(1,28) | 4,75*(2,06) |
| Propensity to take risks | 4,80*(1,09) | 5,07 (1,03) | 6,00*(1,88) | 7,15*(1,75) |
| Responsibility | 6,00 (0,70) | 5,00 (2,00) | 4,60 (1,79) | 5,58 (2,22) |
| Social courage | 4,80 (2,38) | 4,00*(1,77) | 4,62 (2,36) | 7,75*(1,55) |
| Sensitivity | 8,00*(1,22) | 5,07*(1,89) | 3,56*(1,9) | 5,72*(2,51) |
| Anxiety | 4,20*(1,09) | 7,61*(1,44) | 7,05*(1,94) | 3,68*(1,81) |
| Self-control | 4,80 (1,48) | 4,46 (1,80) | 3,54 (2,01) | 5,06 (2,08) |
| Nervous tension | 4,00*(0,83) | 7,00*(1,44) | 7,00*(1,74) | 4,00*(1,25) |

**Note.** The main effects of the “types of educational environments” factor: sociability *F (2, 69) = 1.68, p ≥ 0.05*; verbal intelligence *F (2, 69) =* ***21.36****, p ≤ 0.01;* *self-confidence F (2, 69) =* ***9.13****, p ≤ 0.01;* excitability F (2, 69) = **19.78,** p ≤ 0.01; tendency for self-assertion F (2, 69) = **23.04**, p ≤ 0.01; propensity to take risks F (2, 69) = **5.68**, p ≤ 0.01; responsibility F (2, 69) = 2.09, p ≥ 0.05; social courage F (2, 69) = **16.84**, p ≤ 0.01; sensitivity F (2, 69) = **11.85**, p ≤ 0.01; anxiety F (2, 69) = **26.49**, p ≤ 0.01; self-control F (2, 69) = 2.35, p ≥ 0.05; nervous tension F (2, 69) = **33.16**, p ≤ 0.01 (significant differences are given in bold). An asterisk (*) marks the groups that differ significantly from each other in terms of the Scheffe correction results.
